# Illness, medical expenditure and household consumption: observations from Taiwan

**DOI:** 10.1186/1471-2458-13-743

**Published:** 2013-08-12

**Authors:** Kuangnan Fang, Chi Ma, Yefei Jiang, Linglong Ye, Benchang Shia, Shuangge Ma

**Affiliations:** 1 Department of Statistics, Xiamen University, Xiamen, China; 2 Beijing Institute of Petrochemical Technology, Beijing, China; 3 Department of Statistics and Information Science, FuJen Catholic University, New Taipei, Taiwan; 4 School of Public Health, Yale University, 60 College ST, New Haven, CT 06520, USA

**Keywords:** Illness, Medical expenditure, Household consumption, Taiwan

## Abstract

**Background:**

Illness conditions lead to medical expenditure. Even with various types of medical insurance, there can still be considerable out-of-pocket costs. Medical expenditure can affect other categories of household consumptions. The goal of this study is to provide an updated empirical description of the distributions of illness conditions and medical expenditure and their associations with other categories of household consumptions.

**Methods:**

A phone-call survey was conducted in June and July of 2012. The study was approved by ethics review committees at Xiamen University and FuJen Catholic University. Data was collected using a Computer-Assisted Telephone Survey System (CATSS). “Household” was the unit for data collection and analysis. Univariate and multivariate analyses were conducted, examining the distributions of illness conditions and the associations of illness and medical expenditure with other household consumptions.

**Results:**

The presence of chronic disease and inpatient treatment was not significantly associated with household characteristics. The level of per capita medical expenditure was significantly associated with household size, income, and household head occupation. The presence of chronic disease was significantly associated with levels of education, insurance and durable goods consumption. After adjusting for confounders, the associations with education and durable goods consumption remained significant. The presence of inpatient treatment was not associated with consumption levels. In the univariate analysis, medical expenditure was significantly associated with all other consumption categories. After adjusting for confounding effects, the associations between medical expenditure and the actual amount of entertainment expenses and percentages of basic consumption, savings, and insurance (as of total consumption) remained significant.

**Conclusion:**

This study provided an updated description of the distributions of illness conditions and medical expenditure in Taiwan. The findings were mostly positive in that illness and medical expenditure were not observed to be significantly associated with other consumption categories. This observation differed from those made in some other Asian countries and could be explained by the higher economic status and universal basic health insurance coverage of Taiwan.

## Background

Illness conditions incur medical expense. Multiple studies have shown that medical expenses can have a far-reaching impact on other categories of consumptions [1-4]. The underlying mechanism is simple: with a fixed total budget, when medical expense occurs, individuals and households can be forced to reduce other consumptions, including, for example, food, education, farming expenses, other production means, recreation, and others. Such reductions have been observed in multiple studies [5,6], and may have a significant long-term impact.

**Figure 1:**
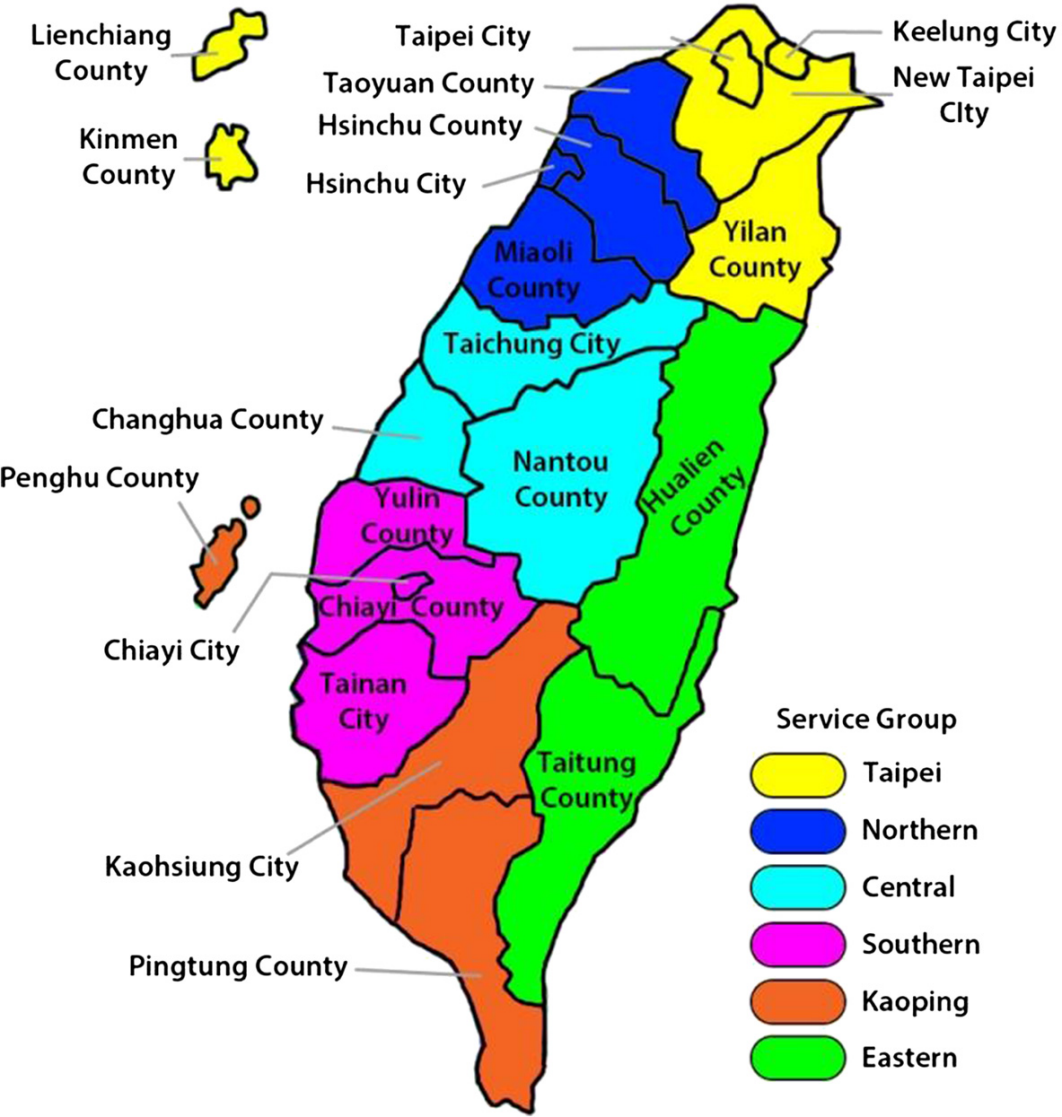
Map of Taiwan.

Taiwan is a state in East Asia. A map is shown in Figure 1. In 2012, the estimated population was about 23 million. It has a relatively higher economic status than many other Asian regions and countries. Its 2011 estimated per capita GDP (PPP) was $37,719, which ranked it 19^th^ in the world and 8^th^ in Asia. Taiwan’s 2011 estimated per capita nominal GDP was $20,100, which ranked it 39^th^. Taiwan is one of the few countries/regions that offer universal health insurance coverage, which has been applauded as one of the most successful and made Taiwan significantly different from most other Asian countries.

In the literature, there are a large number of studies on illness conditions and health sector performance in Taiwan [7-12]. However, our literature review suggested that most studies had been focused on illness conditions and research questions related to health insurance (coverage, cost, effects, etc.). There is a lack of research on the associations of illness and medical expenditure with the overall expenditure structure.

Studies on the relationship between medical expenditure and other types of expenditures have been reported for other Asian countries. For example, the study conducted by Nguyen and others [13] reported the impact of medical expenditure in rural Vietnam, where the per capita income was about $630. It was observed that households with at least one inpatient treatment and/or higher levels of outpatient treatments had significantly reduced consumptions of basic capacities including food, education, and production means. Setboonsarng and Lavado [6] reached a similar conclusion from a study conducted in rural Thailand, where the total household expenditure per year was about $1,723. (Average household size was not reported). The study by Wang and others [5] analyzed a community-based survey conducted in poor, rural areas of mainland China in 2002. It was suggested that “medical expenditure reduced household investment in human capital, physical capital for farm production, and other consumptions that are critical to human well-being” [5].

Our main goal is to provide an updated empirical description of the associations of illness conditions and medical expenditure with other types of expenditures in Taiwan. This objective is similar to that of several published studies [5,13]. However, different from other regions studied in the literature [5,6,13], Taiwan has a much higher economic status and offers universal health insurance coverage. Thus observations are expected to be different, and so this study is warranted beyond the published ones.

## Methods

### Study design and data collection

This study was approved by research ethics review committees at Xiamen University in China and FuJen Catholic University in Taiwan (IRB approval forms available upon request).

The study was designed to cover all major areas of Taiwan. The Taiwan Bureau of National Health Insurance classified the 22 counties and cities into six regions called “service groups” (see Figure 1). The Taipei service group includes Taipei, Keelung New Taipei, Yilan, Kinmen, and Liechiang. The northern group includes Hsinchu County, Hsinchu City, Taoyuan, and Miaoli. The central service group includes Taichung, Nantou, and Changhua. The southern service group includes Tainan, Yunlin, Chiayi County, and Chiayi City. The Kaoping service group includes Kaohsiung, Pingtung, and Penghu. The eastern service group includes Hualien and Taitung.

The survey was conducted by staff at the Data Mining Research Center (DMRC) at Xiamen University in China in June and July of 2012. A Computer-Assisted Telephone Survey System (CATSS) was utilized. Phone numbers used for the survey were obtained from Chunghwa Telecom Co., Ltd., and Taiwan Cellular Co., Ltd., and included both landlines and cell phones. Samples were collected using an RDD (random digit dialing) approach. Specifically, this study used Mitofsky-Waksberg-type samples of active blocks of 100 consecutive phone numbers from all possible such blocks within each city and county [14]. The probability of a block’s initial selection was proportional to the block’s 100 numbers that served individuals and residences. The survey database was updated after each phone call to ensure that no household was sampled more than once.

For each survey, information was first collected to determine inclusion. A household would be excluded if (a) the interviewee refused to participate, (b) the interviewee was less than 18 years old, (c) the interviewee could not provide reliable information on the household, or (d) the interviewee did not live in Taiwan for over six months a year. For each surveyed household, verbal consent was obtained and recorded. The survey included both “snapshot” questions (including, for example, demographic information and insurance status) and “accumulation” questions (such as income and expenses over a period of 12 months prior to the survey). On average, one survey took about ten minutes. Data on 2,607 households was collected. The overall survey response rate was 41.2%.

### Statistical analysis

As “household” remained the basic functional unit for income and expenses, analysis was conducted at the household level. Raw data was examined using various graphical approaches, and no obviously unreasonable measurement was observed. In the first set of analyses, the associations of illness conditions (measured by the presence of chronic disease and inpatient treatment) and medical expenses with household and household head characteristics were examined. The differences between different illness and medical expense groups were quantified using t-tests for continuous variables and chi-squared tests for categorical variables. A similar approach was employed to study the associations between illness and consumptions in the univariate analysis. Multivariate analysis was then conducted, adjusting for the confounding effects of household and household head characteristics. In order to get a comprehensive description, each consumption category was analyzed separately. With a similar strategy, the associations between medical expenditure and other types of consumptions were also analyzed directly. In the analysis of expenditure structure, two sets of analyses were conducted. In the first set, the actual amount of consumption was analyzed. Here, the response variable was continuous, and linear regression was adopted. In the second set of analyses, the percentage of each category of consumption (as of total consumption) was analyzed using logistic-type regression. Model diagnostics were conducted using various graphical tools. The analysis was conducted using R software Version 2.15.1 (http://www.r-project.org).

## Results

### Characteristics of illness conditions and medical expenditure

Table 1 presents summary statistics of household and household head characteristics for the whole cohort and subgroups with different illness conditions and medical expenditure levels. Two measurements were used for illness conditions. The first was the presence of members with chronic diseases, which were long-term and had multiple episodes and low cost per episode. The second was the presence of members with inpatient treatments, which had a low frequency but high cost per episode. There were 1,566 households (60%) with the presence of chronic diseases and 1,540 households (59.1%) with the presence of inpatient treatments in 12 months. Medical expenditure was defined as the per capita, out-of-pocket medical expense accumulated over a period of 12 months. Expenditure data was collected at the household level and then normalized by household size. Taiwan has an effective health insurance system with a low copayment, which has a ceiling and can be completely waived under certain circumstances. Thus, out-of-pocket cost and gross cost can be significantly different. Here, the focus is on out-of-pocket cost, which is a practically more meaningful measure for households. The continuous medical expenses were dichotomized at the median (57,000NT, which is 1,906.63USD under the exchange rate 1USD = 29.851NT) to create “high”- and “low”-cost groups.

**Table 1:** Characteristics of the whole cohort and subgroups with different illness conditions and medical expense levels

		**Presence of chronic disease**		**Presence of inpatient treatment**		**Medical expense**	
**Variables**	**Whole cohort**	**Yes**	**No**	**Yes**	**No**	**High**	**Low**
Total sample	2607	1566	1041	1540	1067	1301	1306
**Data on household**							
Household size	4.72 (1.41)	4.70 (1.40)	4.74 (1.41)	4.73 (1.39)	4.71 (1.43)	4.84 (1.41)	4.60 (1.39)
p-value		0.505		0.669		<0.001	
Younger than 18*							
0	11.12	11.05	11.24	10.71	11.72	9.53	12.71
1	77.37	76.76	78.29	77.41	77.41	78.79	75.96
2	8.21	8.75	7.4	8.7	7.5	8.15	8.27
3	2.57	2.87	2.11	2.53	2.62	2.69	2.45
4	0.46	0.38	0.58	0.52	0.37	0.46	0.46
5	0.23	0.19	0.29	0.19	0.28	0.31	0.15
6	0.04	0	0.1	0	0.09	0.08	0
p-value		0.509		0.726		0.934	
Older than 65*							
0	69.97	70.24	69.55	69.35	70.85	66.95	72.97
1	23.21	22.29	24.59	23.31	23.06	25.44	20.98
2	5.56	5.94	5	5.84	5.15	6.38	4.75
3	0.5	0.7	0.19	0.52	0.47	0.46	0.54
4	0.58	0.64	0.48	0.65	0.47	0.61	0.54
5	0.19	0.19	0.19	0.32	0	0.15	0.23
p-value		0.305		0.465		0.029	
Public insurance coverage*	91.16 (19.14)	91.20 (19.21)	91.09 (19.04)	90.72 (19.82)	91.80 (18.10)	91.36 (18.85)	90.96 (19.43)
p-value		0.881		0.150		0.595	
Private insurance coverage*	90.68 (18.40)	90.46 (18.9)	91.01 (17.5)	90.83 (18.01)	90.46 (18.9)	90.38 (18.4)	90.98 (18.43)
p-value		0.447		0.614		0.403	
Location*							
Taipei	32.72	33.27	31.89	32.47	33.08	33.13	32.31
Northern	16.88	16.91	16.91	17.01	16.68	17.14	16.62
Central	17.38	16.54	18.64	17.27	17.53	17.22	17.53
Southern	14.46	14.81	13.93	14.09	15	14.53	14.4
Kaoping	15.42	15.33	15.56	15.84	14.81	14.83	16
Eastern	3.15	3.19	3.07	3.31	2.91	3.15	3.14
p-value		0.801		0.939		0.973	
I**ncome**	17283	17132	17510	17339	17202.	20497	14082
	(6950)	(7125)	(6674)	(6936)	(6973)	(6846)	(5405)
p-value		0.168		0.621		<0.001	
**Data on household head**							
Age*							
<20	1.76	1.72	1.83	1.95	1.5	1.61	1.91
21-30	8.59	7.6	10.09	7.92	9.56	9.3	7.89
31-40	3.07	3.13	3.98	3.05	3.09	3.46	2.68
41-50	36.59	37.16	35.73	37.08	35.9	37.74	35.45
51-60	39.62	40.36	38.52	39.81	39.36	37.89	41.35
>60	10.36	10.03	10.85	10.19	10.59	9.99	10.72
p-value		0.318		0.691		0.272	
Gender*							
Male	61.80	61.94	61.58	61.69	61.95	60.88	62.71
p-value		0.883		0.925		0.356	
Education*							
<Middle school	18.26	19.16	16.91	18.12	18.46	17.29	19.22
High school	34.79	34.42	35.35	33.77	36.27	35.59	34
Bachelor	38.36	38.38	38.33	39.09	37.3	38.28	38.44
>Bachelor	8.59	8.05	9.41	9.03	7.97	8.84	8.35
p-value		0.354		0.470		0.576	
Marital status*							
Single	16.92	17.11	16.62	16.88	16.96	16.22	17.61
Married	68.66	67.5	70.41	69.55	67.39	69.1	68.22
Divorced	8.17	8.43	7.78	7.73	8.81	8.15	8.19
Widowed	6.25	6.96	5.19	5.84	6.84	6.53	5.97
p-value		0.228		0.505		0.765	
Occupation*							
Government	17.18	16.48	18.25	17.34	16.96	10.91	23.43
State-owned Co	11.93	12.45	11.14	12.6	10.97	9.53	14.32
Private Co	37.63	36.72	39	36.6	34.68	46.66	28.64
Self-employed	19.52	19.86	19.02	18.64	20.81	27.06	12.02
Farmer	8.52	8.43	8.65	8.9	7.97	5.84	11.18
Unemployed	2.07	2.55	1.34	2.14	1.97	0	4.13
Retired	2.57	3	1.92	2.4	2.81	0	5.13
Other	0.58	0.51	0.67	0.39	0.84	0	1.15
p-value		0.152		0.496		<0.001	

Note: Values presented are mean (standard deviation) or percentage for variables marked by *. Income values are in USD.

Table 1 suggests that the distribution of illness conditions is not significantly associated with household characteristics (including household size, number of members younger than 18, number of members older than 65, public health insurance coverage, private health insurance coverage, location, and income) and household head characteristics (including age, gender, education, marital status, and occupation). Such an observation is positive in the sense that socioeconomically less advantaged households – for example, those with lower incomes or living in less developed areas – were not more likely to suffer from illness. The level of per capita medical expense was positively associated with household size (p-value < 0.001) and number of household members older than 65 (p-value 0.029). For example, 66.95% of households in the high expense group had no member older than 65, whereas that percentage was 72.97% in the low expense group. Medical expense was also positively associated with income (p-value < 0.001). Another factor significantly associated with medical expense level was household head occupation. Households with high expenses were more clustered in the “private company” and “self-employed” groups, whereas the low expense group was more evenly distributed. Similar associations between medical expenses and household size, income, and occupation have been observed in the literature [5,9,13].

### Associations of illness conditions with consumption

Nine categories of consumption were considered. More details are available in Table 2. Each category was computed as the per capita consumption accumulated over a period of 12 months. In the preliminary study, we found that households might purchase food, daily goods, clothes, and a few other items together and had trouble recalling the exact cost of each item. Thus, different from some existing studies [5], the category “basic consumption” was created to include food, clothes, production means, utilities, and daily goods. For the whole cohort, the biggest consumption category was basic consumption (30.89%), followed by saving/investment (22.96%), medical expense (15.41%), and insurance (15.34%).

**Table 2:** Per capita consumption of the whole cohort and households with different illness conditions and medical expense levels

		**Presence of chronic disease**		**Presence of inpatient treatment**		**Medical expense**	
**Variables**	**Whole cohort**	**Yes**	**No**	**Yes**	**No**	**High**	**Low**
**Amount of expense (USD)**							
Basic (food, produce, etc.)	3982.5	3934.7	4504.5	3992.1	3968.6	4716.9	3250.9
sd	2020.3	2029.8	2004.7	2045.4	1984.3	2119.8	1612.6
p-value		0.137		0.769		<0.001	
Education	691.0	680.6	706.6	690.8	691.3	819.6	562.9
sd	304.7	306.3	301.8	301.3	309.7	310.2	238.1
p-value		0.033		0.968		<0.001	
Saving/Investment	2960.0	2042.3	2986.7	2978.8	2933.0	3509.6	2412.6
sd	1339.7	1363.3	1303.5	1333.8	1348.4	1370.6	1054.5
p-value		0.403		0.392		<0.001	
Entertainment	689.1	682.4	699.3	691.4	685.9	819.2	559.5
sd	302.4	308.5	292.8	298.4	3-8.1248	307.2	234.3
p-value		0.158		0.649		<0.001	
Insurance	1977.7	1945.9	2025.6	1989.5	1960.7	2345.0	1611.8
sd	1004.6	1008.5	997.3	1006.5	1002.1	1045.0	811.9
p-value		0.047		0.472		<0.001	
Durable goods	398.0	389.7	410.4	400.6	394.1	470.5	325.7
sd	198.4	196.9	200.1	194.9	203.4	205.3	161.8
p-value		0.009		0.415		<0.001	
Alcohol/Tobacco	196.3	193.7	200.1	196.9	195.4	233.8	159.0
sd	100.7	102.7	97.6	101.1	100.1	106.6	78.3
p-value		0.108		0.717		<0.001	
Other	11.8	12.1	11.3	9.1	15.7	4.1	19.5
sd	103.2	109.9	92.4	101.1	106.1	72.2	126.4
p-value		0.841		0.116		<0.001	
**Percentage of expense**							
Basic (food, produce, etc.)	30.89	30.86	30.93	30.82	31.00	30.16	32.01
Education	5.36	5.34	5.39	5.33	5.40	5.24	5.54
Saving/Investment	22.96	23.08	22.79	22.99	22.91	22.44	23.76
Entertainment	5.34	5.35	5.34	5.34	5.36	5.24	5.51
Insurance	15.34	15.26	15.45	15.36	15.31	14.99	15.87
Durable goods	3.09	3.06	3.13	3.09	3.08	3.01	3.21
Alcohol/Tobacco	1.52	1.52	1.53	1.52	1.53	1.49	1.57
Other	0.09	0.10	0.09	0.07	0.12	0.03	0.19

Notes: Basic consumption includes food (rice, meet, vegetable, fruit, etc.), clothes, production means (farming equipment, fertilizer, seed, etc.), utilities (electricity, water, heating, cooking, renting, etc.), and daily goods (toiletries, kitchen supplies, etc.). Education includes tuition, book, and other education-related expenses. Saving/investment include banking and stock. Entertainment includes entertainment, travel, holidays, and other social activities. Insurance: for property, farm product, health, etc. Durable goods include furniture and electronic devices. Alcohol/Tobacco: cigarette, tobacco, wine, and liqueur. Medical expense: outpatient and inpatient services, drugs. Other: expenses not listed above.

The associations between illness conditions and consumptions were investigated using univariate analysis (Table 2) and multivariate analysis (Table 3). In the multivariate analysis, confounding effects of household and household head characteristics were adjusted for (with more details provided in Table 3). Two sets of analyses were conducted. The first set regressed the actual amount of each type of expense (in USD) on illness conditions and confounders, using linear regression. The second set was on the percentage of each category of expense (as of total expense). As the percentage lied between zero and one, logistic-type regression analysis was conducted [15]. Since the number of households that reported “other expense” was small and the actual amount was also small, this expense category was not analyzed.

**Table 3:** Multivariate regression analysis: effects of the presence of chronic disease and inpatient treatment on expense, measured by actual amount (in USD) and percentage

	**Amount of expense**				**Percentage of expense**			
	**Presence of chronic disease**		**Presence of inpatient treatment**		**Presence of chronic disease**		**Presence of inpatient treatment**	
	**Regression coef**	**P-value**	**Regression coef**	**P-value**	**OR**	**P-value**	**OR**	**P-value**
Basic (food, produce, etc.)	−31.038	0.530	−2.133	0.965	0.997	0.847	0.986	0.297
Education	−10.630	0.031	−5.788	0.237	0.991	0.275	0.990	0.204
Medical expense	4.621	0.851	29.990	0.221	1.015	0.285	1.020	0.142
Saving/Investment	19.990	0.420	21.340	0.386	1.010	0.323	1.007	0.469
Entertainment	−1.616	0.744	0.229	0.963	0.999	0.891	0.998	0.784
Insurance	−36.236	0.150	14.187	0.571	0.986	0.299	1.003	0.811
Durable goods	−11.960	0.014	3.455	0.473	0.979	0.102	1.013	0.334
Alcohol/Tobacco	−1.923	0.447	−0.099	0.969	0.980	0.144	0.993	0.594

Notes: Adjusted for household characteristics (size, presence of member(s) younger than 18, presence of member(s) older than 65, basic insurance coverage, commercial insurance coverage, city, urban, and income) and household head characteristics (age, gender, education, occupation, and marital status).

The multivariate analysis suggests that, compared to those without chronic disease, households with chronic diseases had lower levels of expenses on education (an estimated difference of 10.63USD, p-value 0.031) and durable goods (an estimated difference of 11.96USD, p-value 0.014). The presence of inpatient treatment was not significantly associated with expense amount. Both the presence of chronic disease and inpatient treatment were not significantly associated with the percentages of expenses (as of total expense).

### Associations of medical expense with other expenses

Other types of expenses were regressed on medical expense, adjusting for possible confounding effects. More details are provided in Table 4. Two sets of analyses were conducted, with one on the actual amount of consumption and the other on percentage. In the first set of analyses, only the association between medical expense and entertainment expense was statistically significant (p-value 0.049), with an increase in medical expense associated with a small (practically ignorable) increase in entertainment expense. In the analysis of percentage of expense, an increase in medical expense was significantly associated with a decrease in the percentage of basic consumption (with an odds ratio of 0.986, p-value < 0.001), an increase in saving/investment (with an odds ratio of 1.020, p-value < 0.001), and a decrease in insurance (with an odds ratio of 0.974, p-value < 0.001). A significant change in the expenditure structure was observed, although the magnitudes of change measured by the odds ratios were small.

**Table 4:** Multivariate regression analysis: impact of medical expense on other household expenses, measured by both actual amount and percentage

	**Actual amount**		**Percentage**	
	**Regression coef**	**P-value**	**OR**	**P-value**
Basic (food, produce, etc.)	0.025	0.530	0.986	<0.001
Education	−0.003	0.450	0.903	0.239
Saving/Investment	0.012	0.528	1.020	<0.001
Entertainment	0.008	0.049	0.835	0.109
Insurance	−0.026	0.192	0.974	<0.001
Durable goods	−0.002	0.646	0.996	0.169
Alcohol/Tobacco	0.002	0.388	1.000	0.832

Notes: Adjusted for household characteristics (size, presence of member(s) younger than 18, presence of member(s) older than 65, basic insurance coverage, commercial insurance coverage, city, urban, and income) and household head characteristics (age, gender, education, occupation, and marital status).

## Discussion

Our survey showed that a considerable number of households suffered from illness. It is noted that, in this study, the presence of chronic disease and inpatient treatment was measured at the household level. Of the respondents, 59.1% of the households had inpatient treatments, which might seem high. In our preliminary study, we found that very few households had multiple members who had inpatient treatments. As the average household size was 4.72, the percentage of household members who had inpatient treatment could be reasonable. In addition, this inpatient treatment rate is comparable to that in a recent study conducted in the mainland China [16], which has population health characteristics comparable to those in Taiwan.

Consumption patterns observed in this study are significantly different from those in some other published studies. For example, in the study by Wang and others [5], medical expense accounted for 7.9% of the total expense, education accounted for 12%, and saving accounted for 3.8%. In the Vietnam study [13], medical expense accounted for 5.9% of the total expense, insurance accounted for 0.2%, and there was not a separate category for saving. However, the “other” category – which presumably included saving – accounted for only 4.6%. In the Thailand study [6], medical cost accounted for 2% of the total expense, education accounted for 16%, and there was not a separate category for saving. Factors that might contribute to the observed significant differences include the significant differences in total consumption (caused by an overall higher economic status), geographic differences, and rural-urban differences. Households surveyed in this study had a much higher rate of saving/investment. With regular savings, households may be able to cope with expenses caused by health shocks without reducing the cost of daily living [17].

In the analysis of illness conditions, negative associations between the presence of chronic disease and expenses on education and durable goods were observed. The reduction in consumption caused by illness has been observed in multiple studies [4,5,13] and has an intuitive interpretation. However, it should be noted that the extension and magnitude of reduction were much smaller than those observed in published studies. Such a difference can be explained by the significantly higher economic status of Taiwanese households, which can be partly seen from the GDP figures. In the rural China study [5], the total household consumption was 2043.4RMB (less than 400USD; average household size unspecified). In the Vietnam study [13], the average household consumption for a household with 3.8 members was about 740USD. In contrast, in this study, 22.95% of the total household expense was saving/investment. With a high saving rate, households would not need to reduce daily living costs to cope with illness conditions.

In the analysis of actual amount, most other expense categories were found not to be significantly associated with medical expense. This result suggested a relatively stable expenditure structure in response to illness-related financial shocks. The observed insensitivity differs from some published studies [5,13,17-19], in which both positive and negative associations between medical expense and other types of expenses had been observed. Again, such a difference can be explained by the higher income level and higher saving rate of Taiwanese households. Another possible reason is that Taiwan, unlike most regions and countries investigated in the aforementioned studies, offers universal health insurance coverage. The basic health insurance system in Taiwan has been applauded as a successful one. An effective health insurance system can protect households from reducing living costs because of illness. Additionally, the association between medical expense and entertainment was found to be positive and significant. However, the estimated regression coefficient is very small (0.008). We are not able to identify a logical interpretation for this association. We note that this result does not necessarily suggest any causation, and because of the small magnitude, the positive association should not be over-interpreted. The second set of analyses on expense percentage may better describe the scenario with a fixed budget, as the percentages summed to one. Although three significant associations were observed, the magnitudes measured by the odds ratios were small. The observed reductions in basic consumption and insurance were consistent with published studies [5,13]. When the percentage of medical expense increases, those of some other expenses have to decrease. Basic consumption and insurance together account for a big percentage of the total consumption and have room for reduction. The increase in the percentage of saving may reflect people’s risk-averse nature and intention to protect from future health shocks.

### Limitations

The survey response rate was 41.2%, comparable to some published studies conducted in Taiwan [9]. With the phone call survey, we were not able to collect information from those who refused to participate. Thus, we cannot fully verify the “missing at random” condition. This may affect the quality of data. However, “eyeballing” summary statistics in Table 1 does not suggest any systematic bias of our samples. Our brief literature review fails to identify comparable studies to fully validate the quality of our data or otherwise. Ideally, longitudinal data is needed to fully track the changes in non-medical expenditures following illness conditions and estimate the impact of medical expenditure. In this study, only cross-sectional data was available, and some assumptions, such as stationarity, had to be made. An advantage of the phone call survey is that a large number of samples could be collected. However, a drawback is that interviewees had to recall the total amount of income and expenses for a period of 12 months. It has been suggested that such an approach may lead to a biased estimation (usually under estimation) [20]. A collection of other sources of data (for example medical records from hospitals for inpatient treatments) is needed to fully gauge the validity of survey measurements and correct any bias. However, with limited resources, such data was not available, and there is not a good way to fully evaluate the quality of the data. We note that many other survey studies share the same limitation. Illness condition was measured by the presence of chronic disease and inpatient treatment. Such measures did not take into account the type of illness and number of episodes. Of note, similar measures had been adopted in [5,13] and others.

## Conclusion

This study has provided an updated description of illness conditions and medical expenses and their associations with other consumptions. Some of the observations are new and informative. Specifically, studies conducted in other Asian regions and countries, including for example rural mainland China, Vietnam, and Thailand, suggested that illness conditions and medical expenditure had a significant negative impact on household consumption, particularly on basic consumption. In our study conducted in Taiwan, such a negative impact was not observed, and the findings were more positive. In general, in the surveyed population, households were able to cope with illness conditions and medical expenditure using means other than reducing other consumptions. On the other hand, the impact of illness and corresponding cost is still observable. Although the health insurance system in Taiwan has been generally recognized as very effective, there may be still room for improvement to further protect the financial wellness of the insured.

## Competing interests

The authors declare that they have no competing interests.

## Authors’ contributions

SM and BS designed the study. KF, CM, and BS conducted the study. KF, JY and YL conducted statistical analysis. All authors read and approved the final manuscript.

## Pre-publication history

The pre-publication history for this paper can be accessed here:

http://www.biomedcentral.com/1471-2458/13/743/prepub
